# Automatic sleep staging and atonia detection in RBD using single-channel chin EMG

**DOI:** 10.1177/1877718X261468531

**Published:** 2026-08-03

**Authors:** Hans Van Gorp, Jaap F Van Der Aar, Ruud JG Van Sloun, Angelique Pijpers, Pedro Fonseca, Sebastiaan Overeem, Merel M Van Gilst

**Affiliations:** 1 Department of Electrical Engineering, 3169 Eindhoven University of Technology, Eindhoven, 5600 MB, The Netherlands; 2 Philips Sleep and Respiratory Care, Eindhoven, The Netherlands; 3 Sleep Medicine Centre, 3804 Kempenhaeghe Foundation, Heeze, The Netherlands

**Keywords:** REM sleep behavior disorder, RBD, REM sleep without atonia, RSWA, Parkinson's disease, machine learning, automatic sleep staging, deep learning, surrogate sleep staging, EMG

## Abstract

**Study Objectives:**

The diagnosis of rapid eye movement (REM) sleep behavior disorder (RBD) is of clinical interest, as there is a high rate of future conversion to Parkinson's disease and related neurodegenerative disorders. The clinical standard for diagnosis of RBD relies on human scoring of REM sleep without atonia (RSWA) via polysomnography, which is labor-intensive, costly, and unsuitable for long-term monitoring. Existing (semi-)automatic detection methods often depend on prior manual annotation of REM or suffer from low REM classification performance in this population. We here propose a fully automated system using only a single-channel chin electromyography (EMG) for both REM scoring and RSWA quantification.

**Methods:**

We analyzed 485 polysomnographic recordings, including 30 recordings of subjects with RBD. Using the recently introduced factorized score-based diffusion model, we automatically scored REM epochs based on single-channel chin EMG data, and subsequently quantified RSWA using the REM atonia index from literature.

**Results:**

The proposed system demonstrated strong performance in REM scoring in both subjects with and without RBD (REM sensitivity ≥0.80, specificity ≥0.97, positive predictive value ≥0.81). The REM atonia index derived from automatic scoring showed strong agreement with manually scored data, with minimal bias (≤0.03), narrow limits of agreement (≤0.13), and strong linear correlation (Peason's r ≥ 0.88).

**Conclusions:**

Fully automated RSWA quantification using single-channel chin EMG is feasible and performs comparable to semi-automatic methods that rely on human sleep stage scoring based on polysomnography. This single-sensor approach can improve RBD screening accessibility, particularly in ambulatory settings, and facilitate long term monitoring of RSWA.

## Plain language summary title

A new way to detect sleep problems linked to Parkinson’s risk using just one chin sensor

## Plain language summary

Rapid eye movement (REM) sleep behavior disorder is characterized by increased muscle activity during REM sleep, when muscles are normally relaxed. This abnormal activity, known as REM sleep without atonia (RSWA), is currently assessed using overnight sleep studies with many sensors and detailed manual scoring by trained experts. This process is time consuming, expensive, and difficult to apply outside specialized sleep clinics or for long term follow up.

This study investigated whether REM sleep without atonia can be quantified fully automatically using only a single muscle signal recorded from the chin. Instead of relying on expert annotated sleep stages, a machine learning method was used to automatically identify REM sleep from the chin muscle signal alone. During these automatically identified REM periods, muscle activity was quantified using an established metric that is commonly applied in semi automated analyses.

The method was evaluated using nearly five hundred overnight sleep recordings from a clinical population, with thirty recordings coming from individuals diagnosed with REM sleep behavior disorder and the rest from control subjects with other sleep disorders or no sleep disorder. The amount of abnormal muscle activity obtained using automatic REM scoring showed strong agreement with results obtained using standard expert scoring. Differences between the two approaches were small, and large discrepancies that could substantially alter interpretation were rare.

These findings show that REM sleep without atonia can be quantified reliably using fully automatic analysis of a single chin muscle signal, with results that closely align with existing semi automated methods. This work demonstrates technical feasibility and supports the use of simplified sensor setups for automated analysis. The approach may help enable more scalable and accessible RSWA assessment in future clinical and research applications.

## Introduction

The accurate and effective diagnosis of rapid eye movement (REM) sleep behavior disorder (RBD) is of significant interest since RBD is recognized as a prodromal marker for Parkinson's disease and other α-synucleinopathies. Long-term follow-up studies show that over 90% of patients with RBD convert to neurodegenerative diseases.^1–4^ Timely diagnosis of RBD could thus serve as an early warning for the development of neurodegenerative diseases and could aid with scientific research into the mechanisms of disease progression and possible neuroprotective trials.

The current gold-standard for the diagnosis of RBD is based on polysomnography (PSG) annotated by human experts, during which a certain amount of REM sleep without atonia (RSWA) needs to be demonstrated. Following the guidelines of the American Academy of Sleep Medicine (AASM), this quantification consists of two parts.^5,6^ First, as is the case for any sleep study, sleep stage scoring needs to be performed by a human scorer, who requires at the least EEG, EOG, and EMG to be present in the recording. In the case of RBD, the scoring of the REM stage especially needs to be of high quality, since the subsequent analysis is highly dependent on it. Second, the human scorer inspects the chin (and limb) EMG channels during REM to classify whether each REM epoch exhibits loss of muscle atonia, the rules of which are also described in the AASM scoring manual. In addition to the AASM criteria, supplemental scoring guidelines for the assessment of RBD have been published by the International RBD Study Group,^7^ which provide a complementary framework for the clinical evaluation of RBD.

This process has several downsides. The requirement to capture a full PSG entails the use of many different sensors, which is cumbersome for the patient, costly, and impractical for long-term monitoring. Manual annotation of the sleep stages and RSWA is time-consuming, and subject to inter-rater disagreement.^8^ To improve access to RBD screening and long-term monitoring, it is important to simplify the sensor setup. Such simplification inherently demands automation of the analysis, as human scorers need at the least EEG, EOG, and EMG to adhere to the AASM scoring criteria. Automated methods capable of scoring REM and RSWA on a greatly reduced set of sensors could significantly improve the accessibility and scalability of potentially long-term RBD assessment.

There is a large body of literature that has investigated the partial automation of the diagnosis of RBD.^9–16^ These methods typically focus on automatic RSWA quantification and assume that REM stages have already been scored. To that end, manual sleep stage classification is often employed in these studies, resulting only in methods that could be deployed in partially automated systems. These semi-automatic RSWA detectors have the potential to become fully automated if it can be shown that they are robust to REM stages classified by a sleep staging algorithm rather than a human scorer. Cooray et al.^17^ proposed a fully integrated automatic RBD screening tool, demonstrating that it is possible to perform automatic REM scoring using EOG + EMG and subsequent RBD detection from EMG features coupled with REM scoring, thereby dropping the requirement to measure the EEG. However, their method displays fairly low REM sensitivity, averaging below 0.6, which means that it runs the risk of missing critical RSWA epochs and incorrectly identifying the patient as not showing signs of RBD. Furthermore, it was only compared to healthy controls and the sensor setup of both EOG and EMG has the potential to be simplified even further.

In this study, we investigate whether it is possible to develop a fully automated method for RSWA detection using solely a single-channel chin EMG measurement for both REM scoring and RSWA quantification. To that end, we will combine a previously proposed automatic scoring method^18^ for single-channel chin EMG sleep stage scoring with the established REM atonia index (RAI).^9^ We study the performance of the proposed method on a dataset consisting of subjects with RBD versus age- and sex-matched controls with differential diagnoses, including obstructive sleep apnea (OSA), restless legs syndrome (RLS), periodic limb movement disorder (PLMD), and non-REM parasomnias.

We evaluate the method based on two comparisons. Firstly, we calculate the REM scoring performance of our automatic scoring method using single-channel chin EMG in the RBD and control groups by comparing it against ground-truth human sleep stage scoring. We analyze if the REM classification performance is different between the RBD and the control groups and if EMG muscle activity impacts this performance. Secondly, we calculate the RAI on both the automatically scored REM epochs and the human-scored REM epochs, and compare the two using linear correlations, Bland-Altman analysis, and confusion matrices in terms of the final interpretation.

## Methods

### Data

The data used for this study came from the Sleep and OSA Monitoring with Non-Invasive Applications (SOMNIA) dataset^19^ and the HealthBed dataset.^20^ Both datasets consist of overnight PSG recordings performed at Sleep Medicine Center Kempenhaeghe. The SOMNIA dataset consists of overnight recordings from patients at Kempenhaeghe that were made as part of standard clinical care. As such, it constitutes a heterogeneous clinical population, were almost all subjects had one or multiple sleep disorders, and includes subjects diagnosed with RBD. The HealthBed dataset consists of overnight recordings from subjects without any sleep complaints or previously diagnosed disorders. These subjects were explicitly recruited to create a healthy control set.

All recordings were scored by an expert human scorer following the AASM guidelines. In the SOMNIA set, sleep disorder diagnoses were established by certified physicians according to the International Classification of Sleep Disorders, third edition.^6^ Alternative clinical guidelines for RBD assessment, such as those proposed by the International RBD Study Group,^7^ were not explicitly applied or assessed in this study. All recordings made between 2017-01-01 and 2023-10-10, except pediatric recordings, were included. 1347 recordings from this set were used as training input for the automatic sleep stage algorithm used in the present study, explained in further detail in the original work.^18^ In the current study, we used the hold-out test set consisting of 500 recordings. We then excluded all subjects with multiple recordings, yielding 485 test recordings with exactly one recording per subject. This ensured a strictly subject-independent test set, prevented disproportionate influence of individuals with repeated measurements on population-level performance statistics, and guaranteed that the test data were not used to train or tune the sleep staging model.

From the 485 recordings in the hold-out test set, we selected all 30 recordings of subjects with RBD, which constituted the positive group. RBD was often co-morbid with OSA and insomnia, see Table 1. The remaining 455 recordings formed the unmatched control group, which included a variety of sleep disorders. This control group did not match the RBD group in terms of age and sex, as it was generally younger and had a higher proportion of women compared to the RBD group, see Table 2. Therefore, we extracted the largest possible subset of the unmatched control group that was both age- and sex-matched. This matching was done by selecting the *x* closest age and sex matched controls for each RBD recording separately, growing *x* until a Chi-square test on sex or a Mann-Whitney U test on age yielded a significant difference (significance level 0.05). See Supplement A for a more detailed explanation of the matching algorithm. This approach resulted in x=6 matches to each recording in the RBD group, totaling 180 matched control recordings, see Table 2.

**Table 1.** Number of recordings with each sleep disorder diagnosis present in each of the three groups.

Diagnosis	RBD	All controls	Matched controls
Insomnia disorders	8	154	48
Obstructive sleep apnea	15	255	124
Central sleep apnea	1	13	8
Hypoventilation	1	1	0
Narcolepsy	0	9	3
Other hypersomnolence disorders	0	10	1
Insufficient sleep syndrome	0	10	2
Circadian rhythm disorder	0	8	2
NREM Parasomnias	0	25	1
REM Parasomnias except RBD	1	5	1
Other Parasomnias	0	11	4
RLS and/or PLMD	2	57	30
Other movement disorders	0	18	6
Other sleep disorders	0	3	1
No primary sleep diagnosis and/or normal variants	1	16	5
Healthy	0	27	2
RBD	30	0	0
***Total***	***30***	***455***	***180***

*Note that the diagnoses are non-exclusive (except for healthy), e.g., there is a large overlap between subjects diagnosed with insomnia and obstructive sleep apnea. Thus, the columns do not necessarily sum to the total.*

**Table 2.** Demographic details of the RBD group, all non-RBD controls, and the matched control group.

Group	#Recordings	% Women	Age	AHI
RBD	30	20.00	66 [61, 73]	12.95 [4.65, 23.30]
All controls	455	* 40.88	* 52 [38, 61]	11.26 [3.87, 23.29]
Matched controls	180	20.00	63 [57, 68]	18.20 [9.02, 30.77]

*Age and AHI are shown as recording-wise medians with the 25^th^ and 75^th^ percentiles shown between brackets. The control group was matched for age and sex. Differences between the RBD and both control groups were assessed by statistical tests with a significance threshold of p* *=* 0.05. Differences in sex were assessed by a Chi-square test and differences in age and AHI by Mann-Whitney U tests. Significant differences, denoted by a *, were found for sex and age between the unmatched control group and the RBD group.

**Figure 1.**
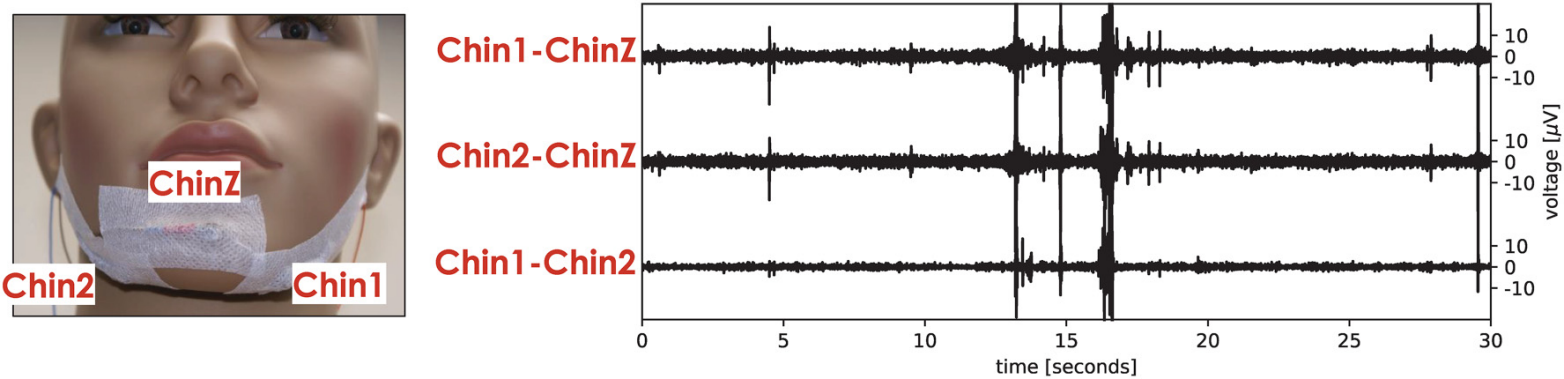
The placement of the chin EMG electrodes follows a unipolar system as shown on the left, where three different derivations are possible. The plot on the right shows the three chin EMG derivations during an epoch of REM sleep of a subject with RBD.

The PSG recordings were made using a standard recording and amplification system (Grael PSG, Compumedics, USA). From these recordings, we extracted the three possible chin EMG signal derivations as shown in Figure 1. According to the AASM manual, the preferred derivation for both sleep staging and RSWA scoring is either of the two electrodes below the mandible (Chin1 or Chin2) referenced to the electrode above the mandible (ChinZ). If ChinZ fails, it should be replaced, or optionally, the derivation between Chin1 and Chin2 can be used as a backup. In this manuscript, we studied the use of all three possible derivations.

The original chin EMG signals were sampled at 512 Hz without any filtering beyond the required analog low-pass filter to sample at the Nyquist rate. These signals are used as input for both the automatic sleep staging algorithm and the automatic RSWA detector, each using slightly different preprocessing. For both applications, we used the preprocessing pipeline detailed in Van Gorp et al..^18^ The only difference is that for the automatic sleep staging algorithm, we resampled the signal to 128 Hz using polyphase filtering and applied a high-pass filter at 10 Hz and a low-pass filter at 49 Hz. For the RSWA detector, we resampled to 256 Hz and filtered between 10 Hz and 100 Hz, with an additional notch filter at 50 Hz. Note that the filter settings for the RSWA detector matched those as recommended by the AASM manual, while those used for the automatic sleep staging algorithm were more restrictive, especially the low-pass filter at 49 HZ instead of the recommended 100 Hz.

Both the SOMNIA and HealthBed studies adhered to the guidelines of the Declaration of Helsinki, Good Clinical Practice, and current legal requirements. Both studies were reviewed by the Maxima Medical Center medical ethical committee, Veldhoven, the Netherlands (SOMNIA: N16.074, HealthBed: NL63360.015.17), by the institutional review board of Philips (Internal Committee on Biomedical Experiments, Eindhoven, The Netherlands) and by the institutional review board of Sleep Medicine Center Kempenhaeghe (Heeze, The Netherlands). The data analysis protocol for this study was approved by the institutional review board of Sleep Medicine Center Kempenhaeghe (Heeze, The Netherlands), reported under CSG.KH.2023.24 and approved on 2023-11-13.

### REM classification

Automatic sleep staging was performed on the three separate single-channel chin EMG signals using the previously trained and validated Factorized Score-based Diffusion Model (FSDM).^18^ The FSDM algorithm was used to obtain 5-class sleep staging, performing epoch-per-epoch classification of Wake, REM, N1, N2, or N3. In the original FSDM publication we reported overall sleep staging performance.^18^ In this manuscript, we focused on the performance of REM stage classification (versus the remaining classes) in relation to the presence of RBD. Therefore, we analyzed REM stage classification performance by comparing the predicted sleep stages to those of expert human scorers. This was evaluated using REM sensitivity, specificity, and positive predictive value (PPV), considering REM as the positive class, and calculated per recording.

We calculated each of these three metrics per recording and subsequently calculated the group-wise median and the 25th and 75th percentiles for each of the three groups (RBD, all controls, matched controls). A metric was sometimes undefined, for example, if no REM sleep was predicted by either the human scorer or FSDM. In such cases, the metric of that recording was not used when calculating the median and percentiles.

To assess if there were any differences in REM classification performance, we performed an exploratory statistical analysis. We compared the performance per recording between the RBD group and either all controls or the matched control group for each channel individually, using the Mann-Whitney U test with a significance threshold of 0.05 and a Bonferroni correction of 6.

Additionally, to quantify the uncertainty in REM classification performance for the RBD group, we performed a non-parametric bootstrap analysis at the recording level. Per-recording values of REM sensitivity, specificity, and PPV were resampled with replacement for 1000 bootstrap iterations, with each iteration drawing 30 recordings, equal to the original sample size. For each resample, the median performance metric was computed. Ninety-five percent confidence intervals were obtained using the percentile method based on the empirical bootstrap distribution.

### RSWA quantification

To perform automatic RSWA detection, we used the REM Atonia Index (RAI),^9^ which is calculated in five steps. First, the chin EMG signal is divided into 1 s mini-epochs. For each mini-epoch, the average rectified amplitude is calculated as follows:

$${\mathrm{rEMG}}_{t}=\frac{1}{\;f_{s}}\sum_{i=0}^{\;f_{s}-1}\left|{\mathrm{EMG}}_{t\cdot f_{s}+i}\right|,$$

Where *t* is the time in seconds, $f_{s}$ the sampling rate, |.| denotes the absolute value, $\mathrm{EM}G_{j}$ the *j* -th sample of the EMG signal, and ${\mathrm{rEMG}}_{t}$ denotes the rectified EMG at time *t*. Second, to remove the background chin EMG power, the minimum amplitude in a window of ± 30 s is subtracted from the average amplitude signal (regardless of sleep stage):

$${\mathrm{cEMG}}_{t}={\mathrm{rEMG}}_{t}-\min(rEMG_{t-30},\ldots ,rEMG_{t+30})\;,$$

where ${\mathrm{cEMG}}_{t}$ is the corrected average amplitude signal. Third, Third, each mini-epoch is classified as belonging to 1 of 3 classes, namely: low muscle activity ( cEMG< 1 μ V), ambiguous muscle activity (1 μ V ≤cEMG≤ 2 μ V), and high muscle activity ( cEMG> 2 μ V). Fourth, we keep only those mini-epochs that were scored as REM, in our case either by the ground truth human scorer or by the automatic scoring method. Fifth, the RAI index is calculated as the proportion of low muscle activity mini-epochs among all non-ambiguous mini-epochs:

$$\mathrm{RAI}=\frac{\sum_{t}\;[{\mathrm{cEMG}}_{t}<1\mu V]}{\sum_{t}\;[{\mathrm{cEMG}}_{t}<1\mu V]+\;[{\mathrm{cEMG}}_{t}>2\mu V]},$$

where [.] denote Iverson brackets, which equate to one if the condition inside of it is met.

RAI values below 0.8 indicate strongly altered levels of atonia during REM sleep and are highly indicative of RBD; values between 0.8 and 0.9 are classified as ambiguous; and values above 0.9 indicate normal levels of atonia during REM sleep. If no REM stages are predicted, the RAI is set to ‘undefined’.

To investigate the influence of muscle activity levels on REM staging performance, we repeated the analysis of the performance metrics (sensitivity, specificity, PPV) using an epoch-level approach. For each manually scored REM epoch, we counted the number of high-activity mini-epochs it contained (i.e., the number of mini-epochs with ${\mathrm{cEMG}}_{t}>2\mu V$ ) and stratified epochs into the following activity bins: 0, 1–5, 6–10, 11–15, 16–20, 21–25, and 26–30 high-activity mini-epochs. Performance metrics were then calculated separately within each bin. To ensure robust estimates, a recording contributed to a given bin only if it contained at least 10 REM epochs in that category.

Finally, to evaluate the impact of manual versus automatic REM scoring on RAI calculation, we compared the resulting values across the three groups (RBD, all controls, matched controls) using scatter plots with a calculation of the linear correlation coefficient (Pearson's r), and Bland-Altman plots with the bias and the 95% limits of agreement. Finally, we compared the RAI values using a confusion matrix based on the four classes as defined in the original paper^9^: RAI<0.8, 0.8 ≤ RAI≤0.9, RAI>0.9, and ‘undefined’. This helped us to evaluate whether any egregious errors occur when using automatic, instead of manual REM scoring. For example, an egregious would occur if the semi-automatic method predicted RAI<0.8 while the automatic method predicted RAI>0.9, resulting in a missed diagnosis.

## Results

### REM classification

The REM staging results for Chin1-ChinZ are shown in Table 3. Supplement B shows the same metrics for the Chin2-ChinZ and Chin1-Chin2 channels. For all groups and all chin EMG signals, the resulting metrics indicated strong performance. However, significant differences were found in REM sensitivity between the RBD group and the all control group across all three chin EMG channels, and even for the matched control group for Chin2-ChinZ (see Supplement B). This highlights the difficulty in identifying all REM epochs in the RBD group, resulting in a higher false negative rate. However, the average REM sensitivity was still ≥0.81 when using Chin1-Chinz for the RBD group (sensitivity was ≥0.79 and ≥0.83 for Chin2-ChinZ and Chin1-Chin2, respectively). Additionally, no significant differences were found in REM specificity or REM PPV for all three channels, indicating that the presence of RBD does not necessarily lead to a higher number of false positives.

**Table 3.** REM classification performance.

Group	Sensitivity	Specificity	PPV
RBD	0.81 [0.73, 0.89]	0.98 [0.95, 0.99]	0.83 [0.75, 0.89]
All controls	* 0.90 [0.81, 0.95]	0.97 [0.95, 0.99]	0.81 [0.70, 0.90]
Matched controls	0.88 [0.79, 0.94]	0.98 [0.96, 0.99]	0.81 [0.69, 0.90]

*Recording-wise medians [25^th^, 75^th^ percentiles] of REM sensitivity (recall), specificity, and PPV for each of the three groups using the Chin1-ChinZ signal. An exploratory statistical test was performed. The performance metrics of all controls and matched controls was compared to RBD using the Mann-Whitney U test (significance level of p = 0.05, Bonferroni correction of 6), revealing significant differences in REM sensitivity only for the all controls group.*

Uncertainty in REM classification performance for the RBD group was quantified using recording-level bootstrapping, as described in the Methods. The resulting 95 percent confidence intervals were [0.74, 0.88] for sensitivity, [0.96, 0.99] for specificity, and [0.78, 0.86] for PPV, indicating that overall REM classification performance in the RBD group was estimated with reasonable precision despite the limited sample size.

**Figure 2.**
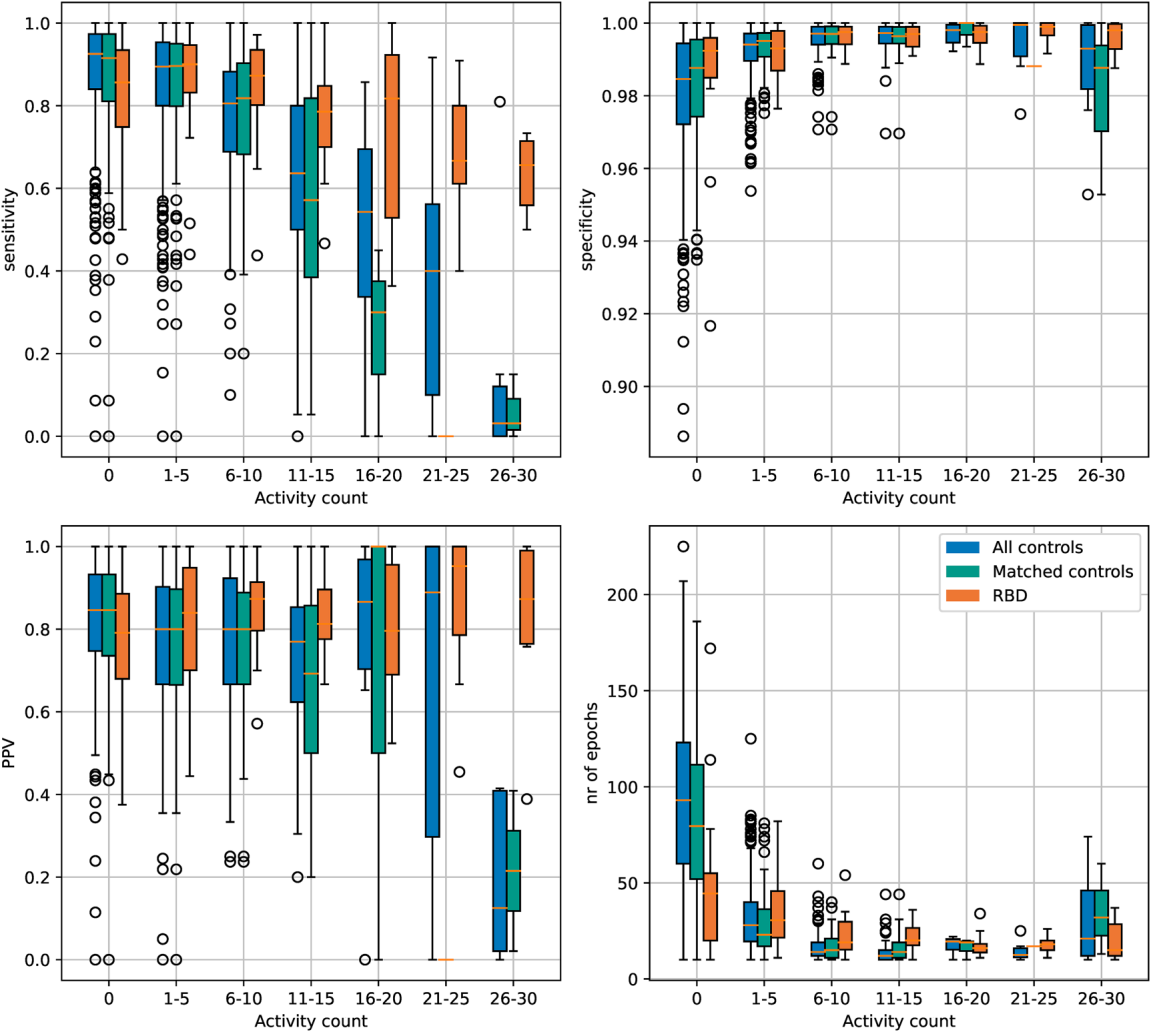
REM sensitivity, specificity, and PPV using the ‘Chin1-ChinZ’ EMG stratified by number of high-activity mini-epochs occurring in an epoch. Boxplot range is taken over recordings. Subplot on the lower right displays the number of epochs belonging to each bin.

To further investigate the source of the observed sensitivity differences, we repeated the analysis by stratifying the REM epochs based on their level of muscle activity, quantified as the number of high-activity mini-epochs within each 30-second REM epoch. Results for Chin1-ChinZ are shown in Figure 2, with additional similar results for Chin2-ChinZ and Chin1-Chin2 available in Supplement B.

Across all groups, we observed a clear decrease in REM sensitivity as the amount of muscle activity within an epoch increased. This effect was particularly pronounced in the control groups, where sensitivity dropped more steeply for epochs with high activity. For the RBD group, sensitivity also decreased with increasing activity, but the decline was less substantial. Notably, even in the bin with *no* high-activity mini-epochs (0-activity), the RBD group still showed a lower REM sensitivity than the controls, which also contributes to the overall lower sensitivity observed for RBD. At the same time, specificity remained consistently very high across all activity levels and for all groups, and the PPV for the RBD group remained stable and high, even in epochs with substantial muscle activity.

### RSWA quantification

**Figure 3.**
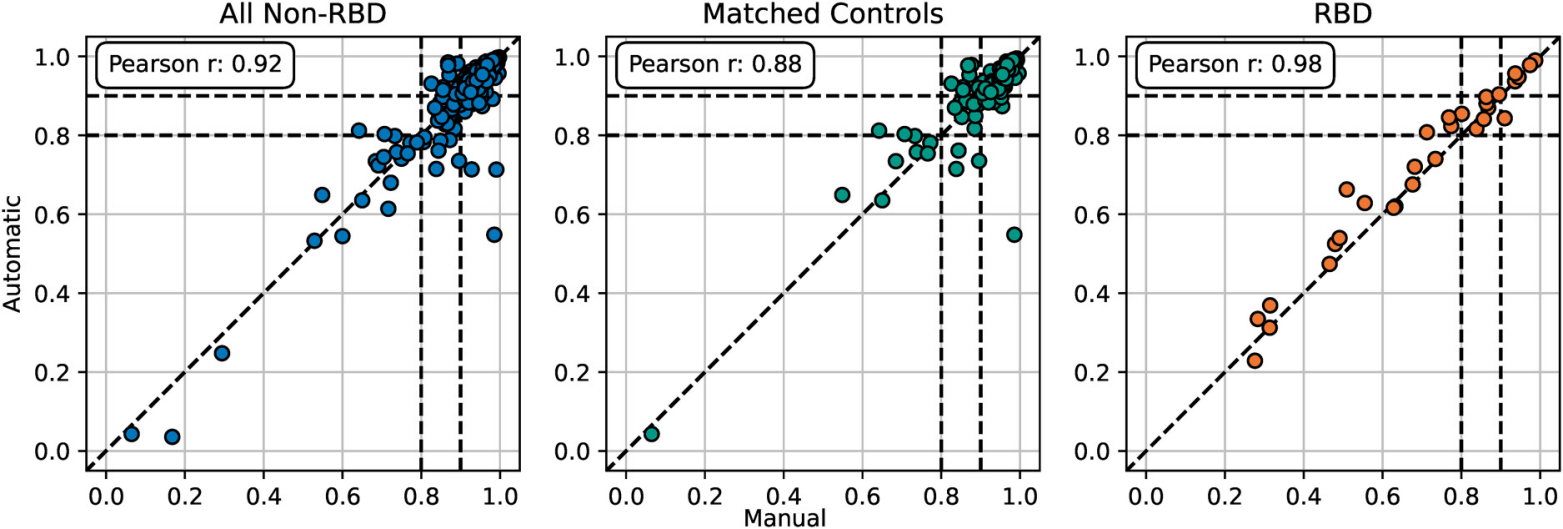
Scatter plots comparing the derived RAI values from the ‘Chin1-ChinZ’ EMG signal using both manual and automatic scoring for the three groups. The manual scoring was based on the full PSG according to the AASM criteria, while the automatic scoring was based solely on the ‘Chin1-ChinZ’ EMG signal. Text displays show the linear correlation coefficient (Pearson's r).

**Figure 4.**
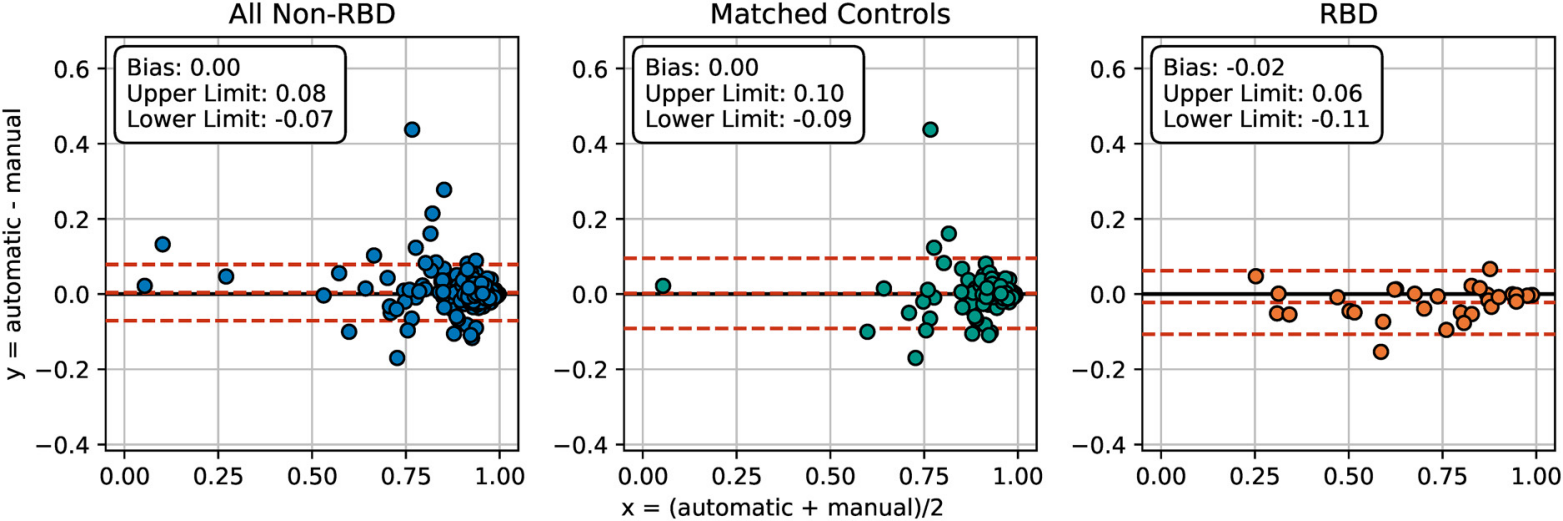
Bland-Altman plots comparing the derived RAI values from the ‘Chin1-ChinZ’ EMG signal using both manual and automatic scoring for the three groups. The manual scoring was based on the full PSG according to the AASM criteria, while the automatic scoring was based solely on the ‘Chin1-ChinZ’ EMG signal. Text displays show the bias and the 95% limits of agreement.

The scatter and Bland-Altman plots comparing the RAI derived from manually scored REM versus automatically scored REM for the Chin1-ChinZ channel are shown in Figures 3 and 4 respectively. From these two plots, it can be observed that there is a strong match between the RAI derived from epochs based on manual and automatic scoring. The linear correlation coefficient was at least 0.92, 0.88, and 0.98 for all controls, the matched controls, and the RBD group respectively. Additionally, there is very low bias across the three groups, and the 95% limits of agreement are also very small, with the highest absolute values being 0.02 for the bias and 0.11 for the limits of agreement.

To again quantify uncertainty, we performed a bootstrap analysis on the RBD group (1000 samples), yielding 95 percent confidence intervals of [-0.04, 0.01] for the bias, [0.04, 0.09] for the upper limit of agreement, and [-0.14, −0.07] for the lower limit of agreement.

**Figure 5.**
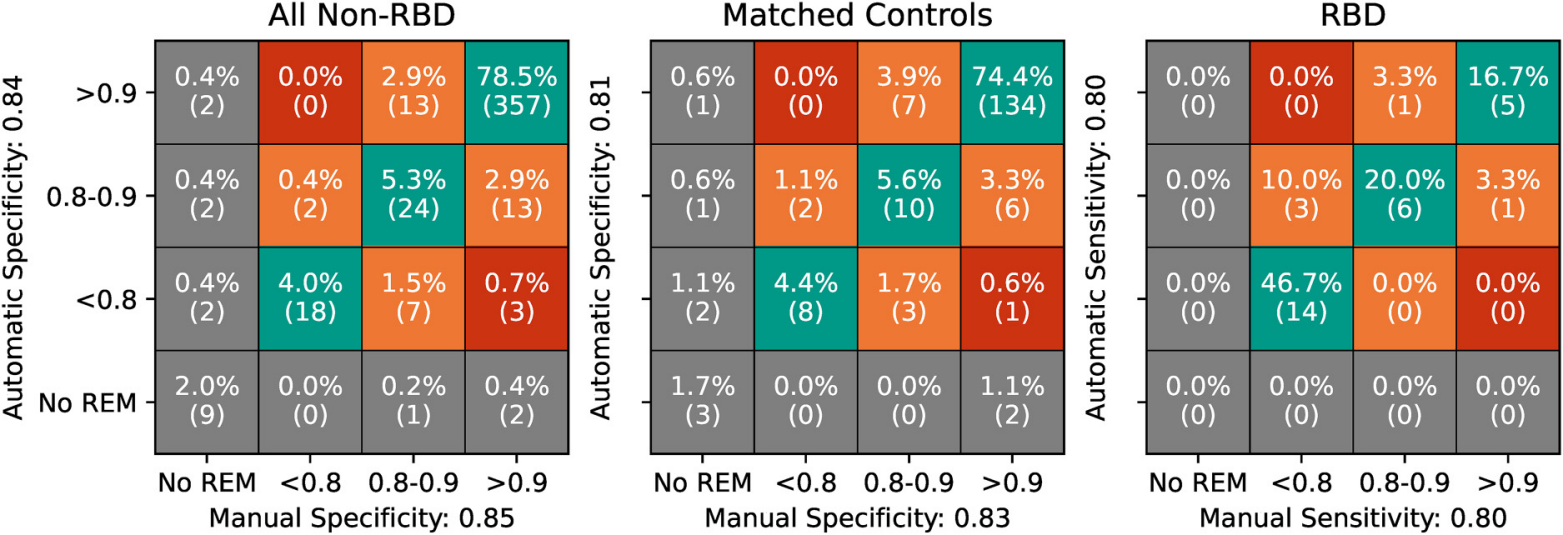
Subject-level confusion matrices comparing the derived RAI values from the Chin1–ChinZ EMG signal using both manual and automatic REM scoring for the three groups. Each matrix entry represents the proportion of recordings (one recording per subject) falling into each category. The manual scoring was based on the full PSG according to the AASM criteria, while the automatic scoring was based solely on the Chin1–ChinZ EMG signal. The ‘No REM’ class was assigned if no REM sleep was detected at all by either the manual or automatic scoring. Percentages are given as a fraction of the total number of recordings (subjects) within each group. The colors indicate the type of deviation between manual and automatic scoring: grey, no REM detected; green, perfect agreement; orange, minor error; red, major error. The sensitivity and specificity were calculated using an RAI cut-off of 0.9, below which recordings were considered positive and above which recordings were considered negative. If no REM was predicted, the recording was not included in the calculation.

Figure 3 also shows the decision boundaries described in literature, namely RAI<0.8, 0.8 ≤ RAI≤0.9, RAI>0.9, with the corresponding confusion matrices for classification based on these thresholds displayed in Figure 5. From these figures, it can be observed that only a small number of recordings fall on opposite sides of the decision boundaries when comparing manual and automatic REM scoring.

The most consequential discrepancies occur when one method classifies a recording as RAI<0.8 and the other as RAI > 0.9, potentially leading to different interpretations. We will refer these to as major errors. In contrast, when one method yields an ambiguous RAI value (0.8–0.9) and the other does not, the discrepancy is considered only a minor error here, as it is less likely to alter the overall conclusion, since one of the outcomes is considered inconclusive.

From Figure 5, it can be observed that major errors were especially rare, accounting for 0.6% in both controls and matched controls, but more importantly, none (0.0%) in the RBD group. Minor errors were slightly more common, accounting for 7.7%, 10.0%, and 16.7%, respectively.

The sensitivity and specificity achieved for the RAI detector were highly similar for both manual and automatic REM scoring. Furthermore, the obtained values are in line with those observed in the literature.^21,22^ For example, previous work has reported 95% confidence intervals of 0.76–0.94 for sensitivity and 0.71–0.90 for specificity when using the RAI for RBD detection.

The same analysis in terms of scatter plots, Bland-Altman plots, and confusion matrices for the Chin2-ChinZ and the Chin1-Chin2 EMG channel are shown in Supplement C. These alternative channels showed the same overall patterns as observed for Chin1-ChinZ. However, the diagnostic accuracy for Chin1-Chin2 was lower in the RBD group for both manual and automatic scoring. This is likely explained by the fact that the RAI detector was developed for Chin1-ChinZ or Chin2-ChinZ, which form mirror configurations, whereas Chin1-Chin2 captures a different underlying biopotential. See also Figure 1 for the difference in electrode placement.

## Discussion

In this manuscript, we investigated whether REM sleep can be reliably scored in subjects with RBD using an automated method based solely on a single-channel chin EMG measurement. We also assessed whether this approach affects downstream RSWA quantification via the REM Atonia Index (RAI), compared to traditional manual scoring based on full PSG.

The overall REM scoring results indicated good performance across all groups and channels. For instance, using the Chin1–ChinZ channel in the RBD group, the automated method achieved a median REM sensitivity, specificity, and PPV of 0.84, 0.98, and 0.83, respectively. However, the sensitivity in the RBD group was significantly lower when compared to controls, highlighting that it is more difficult to detect all REM epochs in these subjects.

Further analysis of REM staging performance, stratified by the level of muscle activity within each REM epoch, provides additional insight into the lower REM sensitivity observed in the RBD group. Across all groups, REM sensitivity decreased as the number of high-activity mini-epochs within an epoch increased, with the steepest decline occurring in the control groups. For the RBD group, sensitivity also decreased with higher activity, but the reduction was less pronounced. Notably, even in epochs with no high-activity mini-epochs, the RBD group still exhibited lower sensitivity than controls, which also contributes to the overall group-level difference. At the same time, specificity remained consistently very high across all activity levels for all groups, and the PPV for the RBD group remained stable and high, even in epochs with substantial muscle activity.

Taken together, these findings suggest that the reduced overall REM sensitivity in the RBD group is driven by two factors. First, individuals with RBD have a much higher proportion of high-activity REM epochs, for which REM sensitivity is lower for *all* groups. Second, even in low-activity epochs, the RBD group shows a modest reduction in sensitivity compared to controls. Importantly, however, the comparatively smaller drop in sensitivity for the RBD group in high-activity epochs indicates that the scoring algorithm partially compensates for the presence of RBD-related muscle activity. In other words, although elevated muscle tone makes REM detection more challenging in general, the method maintains relatively stronger performance for RBD subjects specifically in these high-activity conditions.

A remaining open question is why sensitivity is lower for RBD subjects even in low-activity epochs. Although age, sex, and to some extent AHI were controlled for, unmeasured factors or subtle EMG differences may still contribute. It is also possible that the RAI detector is not sufficiently sensitive to small but physiologically relevant variations within these low-activity REM periods. We leave a more thorough investigation into this effect to future research.

Because automatic sleep scoring algorithms are trained and evaluated on sleep stage scoring performed by humans, the inter-rater agreement of human scorers on RBD effectively limits upper performance. Lower levels of human inter-rater agreement may therefore also be an explaining factor for the lower REM sensitivity in RBD. Unfortunately, inter-rater agreement for REM scoring specifically in RBD has not been systematically studied. However, earlier studies from 2000 and 2004 involving patients with Parkinson's disease which used the R&K scoring manual^23^ reported elevated levels of disagreement among scorers compared to other clinical populations.^24,25^ Direct comparisons between those findings and the present study are challenging due to differences in scoring standards, populations, and methodologies. To better understand the potential impact of human scoring variability on automated REM detection in RBD, new studies with the AASM manual that specifically assess inter-rater agreement for REM staging in this population are needed.

To determine whether REM staging performance using single-channel EMG was sufficient for application to automatic RSWA analysis we analyzed the agreement between the RAI derived from it to the RAI derived from the REM epochs as determined by the gold-standard human reference scoring. We found high levels of agreement between the two. The scatter plots showed high levels of agreement in terms of the linear correlation coefficient. The Bland-Altman plots for all three groups indicated minimal to no bias and narrow limits of agreement. The confusion matrices revealed similarly high levels of agreement, with major errors being exceptionally rare and minor errors occurring at a low rate. This indicates that the lower REM sensitivity in the RBD group has only minimal final decision consequences. The results show that automatic RSWA quantification using the RAI can be performed using fully automatic scoring based solely on single-channel chin EMG that is consistent with the RAI calculated using sleep staging performed by human experts. An additional analysis isolating the impact of REM-epoch misclassification type on RAI computation is provided in Supplement F, showing that further gains in performance are possible primarily through improvements of REM sensitivity.

The implementation of a fully automated method for REM and RSWA quantification has significant clinical implications. By using only single-channel EMG in combination with automatic scoring, RSWA quantification can become much cheaper and more widely accessible. This opens the door to applications in unobtrusive, wearable devices, enabling long-term monitoring in home settings. Such an approach could support treatment and progression monitoring in Parkinson's disease and assist with studies aimed at evaluating interventions, including movement therapy, symptom-suppressing medication, or neuroprotective strategies. RSWA quantification in this regard can be especially useful, considering that increased RSWA has been found to correlate with worsening of cognitive and motor functions in Parkinson's disease.^26^

There are several limitations to this study. Firstly, the data used in this study came from a single sleep center, which may limit the generalizability of our findings to other sleep clinics. Secondly, detailed clinical characterization of the RBD cohort was limited, as information on disease duration, medication use, and individual RSWA event locations was not available in the research dataset. As a result, generalizability across specific RBD etiologies cannot be fully assessed. We were, however, able to retrospectively stratify the cohort into isolated and secondary RBD cases (Supplement D), for which no differences in algorithm performance were observed. Thirdly, the sample size of the RBD group was relatively small. Future research should consider evaluating the automatic method in a larger cohort of subjects, coming from multiple sleep centers. On the technical side, all recordings in this study were made using the exact same recording equipment at a tertiary sleep clinic where the signal quality is continuously monitored. The use of different recording devices and the potentially lower signal quality in ambulatory settings was not considered. Future research could consider studying the impact that these effects might have on the final RSWA quantification.

Of note is that some recordings in the RBD group displayed ‘normal’ amounts of REM atonia (RAI>0.9). For these cases, both the manual and automatic REM scoring classified these as ‘normal’ (RAI>0.9) or as ‘ambiguous’ (0.8 ≤ RAI≤0.9). The reverse was also true, were some of the control group, where some recordings in the control group displayed suspicious amounts of atonia (RAI<0.8). This highlights that the RAI itself has shortcomings concerning RBD detection and not the change from manual to automatic REM scoring. Other RSWA metrics are available in the literature,^9–15^ and the proposed method is agnostic to which specific RSWA metric is applied. Future work could consider the application and comparison of different RSWA metrics. However, in a review paper comparing the performance of different RSWA metrics, their performance was found to be variable across different configurations and comparisons and the authors stated that: “Nevertheless, RAI seems the most sensible one for RBD detection”.^27^

This study demonstrated the feasibility of a fully automatic method for RSWA quantification using single-channel chin EMG. The strong agreement, both in terms of REM staging performance and RSWA quantification, with human expert scored sleep stages indicates that the fully automatic method can be implemented without any loss of performance with respect to the semi-automatic method. This allows for the use of only single-channel EMG as opposed to the full PSG. This approach could enhance the accessibility of RBD screening and enable the monitoring of disease progression with potential interventions in ambulatory settings.

## Supplemental Material

sj-docx-1-pkn-10.1177_1877718X261468531 - Supplemental material for Automatic sleep staging and atonia detection in RBD using single-channel chin EMG Supplemental material, sj-docx-1-pkn-10.1177_1877718X261468531 for Automatic sleep staging and atonia detection in RBD using single-channel chin EMG by Hans Van Gorp, Jaap F Van Der Aar, Ruud JG Van Sloun, Angelique Pijpers, Pedro Fonseca, Sebastiaan Overeem and Merel M Van Gilst in Journal of Parkinson's Disease
