# Supplementary material for: Automatic sleep staging and atonia detection in RBD using single-channel chin EMG
Source: J Parkinsons Dis. 2026 Aug 3;16(6):1324–35. doi: 10.1177/1877718X261468531 (PMC13438777; doi:10.1177/1877718X261468531)
Supplement: Supplementary file 1 [file sj-docx-1-pkn-10.1177_1877718X261468531.docx]

# Appendix A – Matching Algorithm

**Algorithm A1**. Matching algorithm.

**Input:**

List of $N$ RBD recordings: $L_{RBD}\leftarrow\left[ r_{1},r_{2},\ldots,r_{N} \right]$,

List of $M$ unmatched controls: $L_{all controls}\leftarrow\left[ c_{1},c_{2},\ldots,c_{M} \right]$

Significance level $\alpha$

*// keep growing* $x$ *until we find significant differences*

**For** $x=1$ **to** $\left\lfloor M/N \right\rfloor$ **do**

*// initialize lists of still matchable and already matched controls*

$L_{matchable controls}\leftarrow L_{all controls}$

$L_{matched controls}\leftarrow\left[ \right]$

*// loop over all RBD recordings*

**For** **each** $r$ **in** $L_{RBD}$ **do**

*// find x matches per recording*

**For** $i=1$ **to** $x$ **do**

$\Delta sex, \Delta age\leftarrow\infty, \infty$

*// loop over the matchable controls*

**For** **each** $c$ **in** $L_{matchable controls}$ **do**

$\Delta sex here\leftarrow\left| sex\left( c \right)-sex\left( r \right) \right|$ *// sex is encoded as a binary value*

$\Delta age here\leftarrow\left| age\left( c \right)-age\left( r \right) \right|$

*// check if this is a better match*

**If** $\Delta sex here\leq\Delta sex$ **and** $\Delta age here\leq\Delta age$ **do**

$\Delta sex, \Delta age\leftarrow\Delta sex here, \Delta age$

$match\leftarrow c$

**End if**

**End for**

*// add and remove the best match from lists*

Remove $match$ from $L_{matchable controls}$

Add $match$ to $L_{matched controls}$

**End for**

**End for**

*// now check for significance*

$p_{sex}=\mathrm{ChiSquareTest} \left( sex\left( L_{matched controls} \right), sex\left( L_{RBD} \right) \right)$

$p_{age}=\mathrm{MannWhitneyUtest}\left( age\left( L_{matched controls} \right), age\left( L_{RBD} \right) \right)$

**If** $p_{sex}\geq\alpha$ **or** $p_{age}\geq\alpha$ **do**

**Break**

**End if**

**End for**

**Output:**

List of $x\cdot N$ matched controls: $L_{matched controls}$

# Appendix B – REM Scoring with other Channels

**Table B1.** REM classification performance for Chin2-ChinZ.

| **Group** | **Sensitivity** | **Specificity** | **PPV** |
| --- | --- | --- | --- |
| RBD | 0.79 [0.73, 0.89] | 0.97 [0.95, 0.99] | 0.82 [0.74, 0.92] |
| All controls | * 0.90 [0.81, 0.95] | 0.97 [0.95, 0.99] | 0.81 [0.70, 0.90] |
| Matched controls | * 0.88 [0.79, 0.95] | 0.97 [0.96, 0.99] | 0.81 [0.67, 0.89] |

Recording-wise medians [25^th^, 75^th^ percentiles] of REM sensitivity (recall), specificity, and PPV for each of the three groups using the Chin2-ChinZ signal. The performance metrics of all controls and matched controls was compared to RBD using the Mann-Whitney U test (significance level of p=0.05, Bonferroni correction of 6), revealing significant differences in REM sensitivity.

**Table B2.** REM classification performance for Chin1-Chin2.

| **Group** | **Sensitivity** | **Specificity** | **PPV** |
| --- | --- | --- | --- |
| RBD | 0.83 [0.74, 0.87] | 0.98 [0.95, 0.99] | 0.81 [0.72, 0.91] |
| All controls | * 0.90 [0.80, 0.95] | 0.97 [0.95, 0.99] | 0.81 [0.71, 0.90] |
| Matched controls | 0.89 [0.79, 0.95] | 0.98 [0.96, 0.99] | 0.82 [0.70, 0.91] |

Recording-wise medians [25^th^, 75^th^ percentiles] of REM sensitivity (recall), specificity, and PPV for each of the three groups using the Chin1-Chin2 signal. The performance metrics of all controls and matched controls was compared to RBD using the Mann-Whitney U test (significance level of p=0.05, Bonferroni correction of 6), revealing significant differences in REM sensitivity only for the all controls group.

**Figure B1.** REM sensitivity, specificity, and PPV using the ‘Chin2-ChinZ’ EMG stratified by number of high-activity mini-epochs occurring in an epoch. Boxplot range is taken over recordings. Subplot on the lower right displays the number of epochs belonging to each bin.

**Figure B2** REM sensitivity, specificity, and PPV using the ‘Chin1-Chin3’ EMG stratified by number of high-activity mini-epochs occurring in an epoch. Boxplot range is taken over recordings. Subplot on the lower right displays the number of epochs belonging to each bin.

# Appendix C – RSWA Quantification with other Channels

**Figure C1.** Scatter plots comparing the derived RAI values from the ‘Chin2-ChinZ’ EMG signal using both manual and automatic scoring for the three groups. The manual scoring was based on the full PSG according to the AASM criteria, while the automatic scoring was based solely on the ‘Chin2-ChinZ’ EMG signal. Text displays show the linear correlation coefficient (Pearson’s r).

**Figure C2.** Scatter plots comparing the derived RAI values from the ‘Chin1-Chin2’ EMG signal using both manual and automatic scoring for the three groups. The manual scoring was based on the full PSG according to the AASM criteria, while the automatic scoring was based solely on the ‘Chin1-Chin2’ EMG signal. Text displays show the linear correlation coefficient (Pearson’s r).

**Figure C3.** Bland-Altman plots comparing the derived RAI values from the ‘Chin2-ChinZ’ EMG signal using both manual and automatic scoring for the three groups. The manual scoring was based on the full PSG according to the AASM criteria, while the automatic scoring was based solely on the ‘Chin2-ChinZ’ EMG signal. Text displays show the bias and the 95% limits of agreement.

**Figure C4.** Bland-Altman plots comparing the derived RAI values from the ‘Chin1-Chin2’ EMG signal using both manual and automatic scoring for the three groups. The manual scoring was based on the full PSG according to the AASM criteria, while the automatic scoring was based solely on the ‘Chin1-Chin2’ EMG signal. Text displays show the bias and the 95% limits of agreement.

**Figure C5.** Subject‑level confusion matrices comparing the derived RAI values from the Chin2–ChinZ EMG signal using both manual and automatic REM scoring for the three groups. Each matrix entry represents the proportion of recordings (one recording per subject) falling into each category. The manual scoring was based on the full PSG according to the AASM criteria, while the automatic scoring was based solely on the Chin2–ChinZ EMG signal. The ‘No REM’ class was assigned if no REM sleep was detected at all by either the manual or automatic scoring. Percentages are given as a fraction of the total number of recordings (subjects) within each group. The colors indicate the type of deviation between manual and automatic scoring: grey, no REM detected; green, perfect agreement; orange, minor error; red, major error. The sensitivity and specificity were calculated using an RAI cut‑off of 0.9, below which recordings were considered positive and above which recordings were considered negative. If no REM was predicted, the recording was not included in the calculation.

**Figure C6.** Subject‑level confusion matrices comparing the derived RAI values from the Chin1–Chin2 EMG signal using both manual and automatic REM scoring for the three groups. Each matrix entry represents the proportion of recordings (one recording per subject) falling into each category. The manual scoring was based on the full PSG according to the AASM criteria, while the automatic scoring was based solely on the Chin1–Chin2 EMG signal. The ‘No REM’ class was assigned if no REM sleep was detected at all by either the manual or automatic scoring. Percentages are given as a fraction of the total number of recordings (subjects) within each group. The colors indicate the type of deviation between manual and automatic scoring: grey, no REM detected; green, perfect agreement; orange, minor error; red, major error. The sensitivity and specificity were calculated using an RAI cut‑off of 0.9, below which recordings were considered positive and above which recordings were considered negative. If no REM was predicted, the recording was not included in the calculation.

# Appendix D – Isolated and Secondary RBD

**Figure D1.** Scatter plots comparing the derived RAI values from the ‘Chin1-ChinZ’ EMG signal using both manual and automatic scoring for the RBD group, split into isolated RBD and secondary RBD. The manual scoring was based on the full PSG according to the AASM criteria, while the automatic scoring was based solely on the ‘Chin1-ChinZ’ EMG signal. Text displays show the linear correlation coefficient (Pearson’s r).

**Figure D2.** Bland-Altman plots comparing the derived RAI values from the ‘Chin1-ChinZ’ EMG signal using both manual and automatic scoring for the RBD group, split into isolated RBD and secondary RBD. The manual scoring was based on the full PSG according to the AASM criteria, while the automatic scoring was based solely on the ‘Chin1-ChinZ’ EMG signal. Text displays show the bias and the 95% limits of agreement.

**Figure D3.** Subject‑level confusion matrices comparing the derived RAI values from the Chin1–ChinZ EMG signal using both manual and automatic REM scoring for the RBD group, split into isolated RBD and secondary RBD. Each matrix entry represents the proportion of recordings (one recording per subject) falling into each category. The manual scoring was based on the full PSG according to the AASM criteria, while the automatic scoring was based solely on the Chin1–ChinZ EMG signal. The ‘No REM’ class was assigned if no REM sleep was detected at all by either the manual or automatic scoring. Percentages are given as a fraction of the total number of recordings (subjects) within each group. The colors indicate the type of deviation between manual and automatic scoring: grey, no REM detected; green, perfect agreement; orange, minor error; red, major error. The sensitivity and specificity were calculated using an RAI cut‑off of 0.9, below which recordings were considered positive and above which recordings were considered negative. If no REM was predicted, the recording was not included in the calculation.

# Appendix E – Impact of the AHI (in REM)

**Figure E1.** REM sensitivity, specificity, and PPV using the Chin1–ChinZ EMG channel, stratified by the subject’s AHI. Boxplots represent variability across recordings. The lower‑right panel shows the number of recordings in each AHI bin. Overall, REM staging performance did not differ meaningfully across AHI values. The RBD group showed some variation, but the number of recordings per AHI bin was too small to draw reliable conclusions (N = [8, 9, 9, 4]).

**Figure E2.** REM sensitivity, specificity, and PPV using the Chin1–ChinZ EMG channel, stratified by the subject’s AHI in REM. Boxplots represent variability across recordings. The lower‑right panel shows the number of recordings in each AHI bin. Overall, REM staging performance did not differ meaningfully across AHI values. The RBD group showed some variation, but the number of recordings per AHI bin was too small to draw reliable conclusions (N = [10, 11, 4, 5]).

**Figure E3.** RAI for both manual and automatic REM stage scoring using the ‘Chin1-ChinZ’ EMG channel, stratified by the subject’s AHI. Boxplots represent variability across recordings. Overall, the RAI did not differ meaningfully across AHI values. The RBD group showed some variation, but the number of recordings per AHI bin was too small to draw reliable conclusions (N = [8, 9, 9, 4]).

**Figure E4.** RAI for both manual and automatic REM stage scoring using the ‘Chin1-ChinZ’ EMG channel, stratified by the subject’s AHI in REM. Boxplots represent variability across recordings. Overall, the RAI did not differ meaningfully across AHI values. The RBD group showed some variation, but the number of recordings per AHI bin was too small to draw reliable conclusions (N = [10, 11, 4, 5]).

# Appendix F – Impact of FP and FN on the calculated RAI

*Add missed REM*

*Remove wrongfully included REM*

**Figure F1.** RAI calculated on the Chin1-ChinZ EMG electrodes for the REM epochs as classified by the automatic method, manual scoring, and the intersection of the two. As can be observed from the figure, the calculated RAI remains stable.
